# Mental health indicators and their lifestyle associations in German students: a gender-specific multivariable analysis

**DOI:** 10.1186/s12889-022-13777-7

**Published:** 2022-07-25

**Authors:** Lina Spagert, Christian Janssen, Christoph Geigl

**Affiliations:** Department of Applied Social Sciences, Munich University of Applied Sciences, Am Stadtpark 20, 81243 Munich, Germany

**Keywords:** Mental health, Students, Anxiety, Depression, Gender differences, Counselling

## Abstract

**Background:**

Statistics show that the number of received psychosocial counselling sessions remains at a constantly high level or has even increased since the COVID-19 pandemic situation in 2020. The objective of this work is to identify factors associated with students’ mental health to improve prevention and promotion in mental health at universities.

**Methods:**

The analyses were based on a cross-sectional data set collected by an online survey among 1,842 students from a German University of Applied Sciences in 2014. Descriptive statistics as well as nine different multiple linear regression models were calculated with IBM® SPSS® Statistics software. Mental health indicators used were mental health-related quality of life (mental HRQOL), depression, and anxiety, which were analysed in a gender-specific manner.

**Results:**

The analyses showed that the mean of the mental HRQOL score of the SF-36 for the student sample (46.68) was lower than the values for German (48.76) or American (51.34) norm samples. A key finding was the differences in mental health indicators between male and female students. Women reported worse mental health status in comparison to men. Female gender (ß of -.09; *p* < 0.01), age (ß of -1.05; *p* < 0.01), underweight (ß of -.09; *p* < 0.05), smoking (ß of -.10; *p* < 0.05) and drug consumption (ß of -.15; *p* < 0.001) were negatively associated with mental health indicators. In our sample, a moderate consumption of alcohol within the female population (ß of .12; *p* < 0.01) and physical activity within the male sample (ß of .09; *p* < 0.05) were positively associated with mental health indicators.

**Conclusion:**

The gender-specific differences of students’ mental health and its associations could be an important result for counselling services at universities to adjust methods according to gender. Contrary to the general societal perception, students have lower mental health than a norm sample even before the pandemic. Due to the additional mental stress caused by the pandemic, it can be assumed that mental health problems have increased even more. Universities should therefore pay more attention to the mental health of their students.

**Supplementary Information:**

The online version contains supplementary material available at 10.1186/s12889-022-13777-7.

## Background

In Germany, 2,217,604 students enrolled at universities in the winter term of 2010/2011. Ten years later, 2,948,695 people started studying (winter term 2020/2021). This corresponds to an increase of 33% within ten years [1]. Usually, enrolling in university starts a new stage of life for many young people, often in combination with more independence and living in one´s own home. In addition, the transition from being an adolescent to becoming an adult can be challenging. Therefore, between the ages of 16 and 24, there is a higher risk of suffering from mental disorders [2]. According to the report of the Federal Ministry of Education and Research, 23% of students suffer from health restrictions and 11% stated problems in their studies due to health issues. Mental disorders were the impairments mostly mentioned [3]. Due to the pandemic situation researchers found out that 73% of the students reported an increase in mental health issues [4]. It is therefore important to establish offers that improve or prevent the mental health of students. Herrman et al. [5] emphasize the importance of mental health by saying that there is no general health when there is no mental health. Furthermore, they point out that mental health is necessary to cope with everyday challenges and to contribute to the community to maintain a functioning structure.

### Depression

The most common mental illness is depression with differentiated severity from mild to severe. According to the Diagnostic and Statistical Manual of Mental Disorders (DSM-5), the following symptoms may be present with depression: depressive mood or decreased interest for at least two weeks, changes in weight, tiredness, changes in sleep, thoughts of suicide, or changes in appetite. If the episode lasts for more than two years, one speaks of severe depression [6]. In a national online survey among US students, depression was diagnosed in 27.62% of the 27,961 participants [7]. Another study, which analysed 3,706 undergraduate students from seven universities in the UK, comes to a similar conclusion, saying that 22.5% of students suffer from a depressive mood [8]. An analysis of 347 German university students using the Hospital Anxiety and Depression Scale (HADS) reveals that 2.9% of the students have a depression and 6.1% are on the verge of having a depression. Students from law, veterinary medicine, human medicine, theology, and psychology participated in the study. Differentiated by course of study, there is a significant difference with regard to depression: students of theology suffer more frequently from depression than students of human medicine and veterinary medicine [9].

### Anxiety

Substance consumption, diseases or physiological factors can cause anxiety. Anxiety disorders can be differentiated by the reason for anxiety or in which context the disorder appears and are very common in connection with depressive symptoms. According to the DSM-5, the following symptoms may be present with anxiety disorders: palpitation, sweating, shaking, derealisation, shortness of breath, fear of losing control or of dying. [6]

From students who go to a psychotherapeutic counselling center, 56% suffer from test anxiety and 48% report on inexplicable fear and anxiety [10]. According to the Federal Ministry of Education and Research, 12% of the surveyed students are impaired because of exam anxiety [3]. An analysis using the HADS shows that 5.2% of the study participants had a pronounced anxiety and 13.8% were borderline [9].

### Mental health—a gender problem?

In this article, the term gender is used to describe both meanings of sex and gender according to the National Institutes of Health [11]. Hence, gender includes biological differences as well as socially learned behavior. All studies that differentiated between gender found differences in mental health between women and men. Female students generally indicated a poorer mental state of health and a lower level of mental well-being [3, 7, 8, 12–18]. The only exception to this is the alcohol syndrome, which affects men more often than women [13, 17]. In general, there is controversy about the causes of this gender difference. The question remains whether women are more at risk of developing mental disorders or whether the better health awareness of women means that mental illnesses are diagnosed more often, as they are more likely to consult physicians or psychologists. However, it can be certainly assumed that women are at a higher risk of developing psychological problems simply because of their hormonal cycle and its changes during menstruation, after giving birth or during menopause. In addition, women in all social classes and countries are more frequently exposed to domestic and sexual violence than men, which is a severe risk factor for developing a mental disorder [19].

The aim of this analysis is to first give an overview of existing self-reported symptoms of the main mental disorders (mental health-related quality of life [HRQOL], depression, and anxiety) and its associations in German students. Regression analyses were used to identify significant factors that were positively or negatively associated with students' mental health. This information can help to improve the target orientation of interventions regarding mental health prevention and promotion at universities. Thus, higher education institutions can develop better targeted programs to maintain and improve students’ mental health.

So far, mental health indicators as mental HRQOL, depression, and anxiety have not yet been strongly evidenced in German students. Especially their associations with several lifestyle and sociodemographic factors are largely unclear. Moreover, we are not aware of any study that has examined the association between several lifestyle and sociodemographic factors and mental HRQOL, depression, and anxiety among German students in comprehensive statistical models.

A gender-specific understanding of the relationships between lifestyle and sociodemographic factors and mental health indicators based on a sample of German university students may provide important new insights in this specific group. The findings could serve identify groups with social risk profiles that might particularly benefit from interventions to maintain and improve students ‘mental health. Considering lifestyle and sociodemographic factors in targeting interventions may help reduce mental health disorders. The objectives of the analysis were to examine:

1. The mental health status using the indicators mental HRQOL, depression, and anxiety; and
2. Associations between lifestyle and sociodemographic factors and students' mental health indicators in a sample of German university students.

## Methods

A full survey of all 17,524 students at the Munich University of Applied (HM) was conducted from 24.03.2014 to 13.04.2014. The sample comprises 1,842 students, which corresponds to a response rate of 11.4%. Data were collected using an online questionnaire designed on the program Education Survey Automation Suite (EvaSys). The self-administered questionnaire was sent to all students by e-mail via the university's distribution list.

The beginning of the semester was deliberately chosen as survey period, as it can be assumed that students are less motivated to take part in the survey at the end of the semester due to the exam period. In addition a bias could possibly arise in relation to the results on mental health due to the exam stress [20]. The questionnaire began with an information letter about the study’s purpose, how to answer questions, and informed consent to participate in the study.

At the beginning, descriptive statistics were calculated for the relevant variables and then *t*-tests were carried out to detect differences between male and female students. To avoid implausible beta values, limitation of the size of R and poor recognition of the importance of the independent variables, multicollinearity was tested. Multicollinearity occurs when there are strong correlations between two or more independent variables [21]. In total, nine multiple regression models were created to analyse the associations with mental HRQOL, depression, and anxiety; each calculated in addition separately for male and female students. For the multiple regression analyses, the variables were simultaneously integrated into the model using the method “enter”.

For the methodological approach for the sum scores of the SF-36 the results were compared to a German and an American norm sample. The means of the Mental Component Summary (MCS; mental HRQOL) for the German norm sample were 48.76 (SD = 10.65) for women and 51.34 (SD = 9.08) for men. In the American sample, the mean for MCS was slightly higher with 49.84 (SD = 9.43) for women and 51.92 (SD = 8.82) for men [22]. For all analyses, IBM® SPSS® 25 was used and statistical significance levels were defined as *p* < 0.05.

### Measures

#### Mental HRQOL

The subjective state of mental HRQOL was measured using the validated 36-Item Short-Form-36-Health-Survey (SF-36), which mainly assesses the physical, psychological, and social dimensions of HRQOL. Two main scales were calculated, the Physical Component Summary (PCS; physical HRQOL) and the Mental Component Summary (MCS; mental HRQOL). For the purpose of the study, only the mental HRQOL was used, with a minimum of -1.27 and a maximum of 80.74 [23].

#### Depression index

A depression index was created to measure depression symptoms. Therefore, a total of 15 questions were used. The items were similar to the items in the Beck Depression Inventory or described the symptoms of major depression according to the DSM-5 [24]. In some cases, the individual items still had to be recoded so that higher values mean higher depressiveness. Using Cronbach’s alpha as a marker of reliability, variables were removed for safety's sake and a new scale with the following nine items was created: *How often in the last few weeks have you been: full of energy (1); calm and relaxed (2); happy (3); discouraged (4) and sad/exhausted (5)? How badly do you suffer from the following ailments: inner unrest (6); insomnia (7); poor concentration (8); depression (9)?* The 9 items were summed up to create the depression index, which can take on values between 0 and a 37. Higher values indicate higher depressiveness, so a value of 0 would mean that there are no depressive signs.

#### Anxiety

Anxiety was measured using a single item of a modified version of the Zerssen Beschwerdeliste [25]. The score can take values from 1–4, whereby higher scores indicate stronger burden of anxiety, which possibly interferes with daily living, but does not necessarily require an anxiety disorder. The following question was used: *How much do you suffer from anxiety?* (very strong, moderately, slightly, not at all).

#### Nutritional behavior

Previous research examined the connection between food consumption and stress, as well as depression or mental wellbeing [26–28]. In a three-country study, students were interviewed using a Food Frequency Questionnaire (FFQ), the Cohen Perceived Stress Scale, and a modified version of the Beck Depression Inventory in Germany (*n* = 696), Poland (*n* = 489), and Bulgaria (*n* = 654). For the male participants, there was no correlation between the food consumed and the stress level or the depressive symptoms. For the female students, however, the perceived stress correlates with an increased consumption of sweets and fast food as well as a reduced consumption of fruit and vegetables. In addition, depressive symptoms correlate with a low consumption of vegetables, fruit, and meat [29]. A survey with a total of 3,362 Iranians about their food consumption and mental health (using the HADS and the General Health Questionnaire) reveals that women who consumed many fruits were less likely to suffer from depression, anxiety, or psychological stress. The consumption of vegetables correlates with lower levels of depression in women and with fewer anxiety disorders in men. High consumption of fruit and vegetables is associated with lower psychological stress among the male study participants and with fewer symptoms of depression among the female study participants [30]. Therefore, the nutrition index was formed from a total of four questions with the following coding: *How often do you eat sausage or ham in a week? / How often do you eat meat in a week? 4* = *not at all, 3* = *less than once a week, 2* = *one to three times a week, 1* = *three to six times a week, 0* = *daily; How often do you eat fruit in a week? / How often do you eat salad or vegetables in a week? 0* = *not at all, 1* = *less than once a week, 2* = *one to three times a week, 3* = *three to six times a week, 4* = *daily.* The values ​​were added and divided by 4. This means that values ​​between 0 and 4 can be assumed, meaning *0* = *a rather “unhealthy” diet and 4* = *a rather “healthy” diet.*

#### Physical activity

In a controlled trial study, the effect of physical activity on depression was analysed. The 202 study participants were randomly divided into four groups as follows: group 1: physical exercises under supervision; group 2: physical exercises that are carried out at home; group 3: drug treatment with antidepressants; group 4: placebo. The study shows that the effects of exercise brought about the same benefits as the use of antidepressants [31]. The data of 14,706 students were analysed to find associations between physical activity, mental health, stress, and sociability. They found out that students who followed the recommended physical activity had better mental health and felt less stress. Patients who have a clinically relevant diagnosis of mental disorders usually have already had unspecific symptoms years before. On average, patients showed symptoms of depression, anxiety or concentration difficulties five years before a diagnosis with schizophrenia [14]. This pattern of unspecific symptoms appearing years in advance was also found for other mental disorders. Early interventions and methods of treatment showed a positive impact on the progress of mental diseases, whereas late diagnosis can cause chronicity of mental disorders [32].

To measure physical activity two questions were used: 1. *Do you do gymnastics, fitness, or sports? and* 2. *How many hours is that in a week?* Those who did not do any sports a week were given the value 0 for no physical activity. The other students stated in absolute numbers how many hours per week they do sports.

#### Faculty

A study examining the health of German university students found differences between the study courses. Test anxiety is higher among veterinary medicine students than among all other students (human medicine, law, psychology, and theology). There are clear disparities regarding to psychology students. These express psychosomatic complaints significantly more frequently than law students. In addition, psychology students were more likely to report physical symptoms and suffer from anxiety and insomnia more often than medicine students [9].

The faculties were categorized into three departments, comparable to the division of the University of Applied Sciences Munich, namely into technology, economics, and social sciences. The university subdivides also into design, which was added to the social sciences due to the small sample in this group. The gender distribution in the various faculties showed that men mostly complete technical courses (72.9% men vs. 25.9% women), while women were more represented in the social field (83.5% women vs. 16.5% men). More women also studied in the economic field, whereas the ratio was more balanced with 63.4% female and 36.6% male students. In order to be able to calculate a linear regression this categorical variable was provided with a dummy coding. In this case, social science was chosen as the reference category.

#### Alcohol consumption

A long-term study showed the connection between anxiety disorders and alcohol abuse. For this purpose, students in the American Midwest were asked about their mental health status at the beginning of their studies, after one, four and seven years. In total, the data from 454 people interviewed were analysed. It turned out that the likelihood of developing an anxiety disorder or alcohol syndrome was up to five times higher if one of the two disorders was already present. In addition, the likelihood of developing an anxiety disorder increased in the seventh year if there was already an alcohol abuse in years one or four and vice versa [33]. According to the German Nutrition Society [34], the recommended maximum amount of alcohol per day is 10 g for healthy women and 20 g for healthy men. Taking into account the overall mortality rate, no more than 100 g alcohol should be consumed per week [22]. In order to calculate the amount of alcohol per day, an index was created that includes the total amount of alcohol consumed per day. For this purpose, the approximately corresponding alcohol quantities were estimated for the respective answer options for beer, wine, and schnapps *(half a liter beer≈15 g alcohol; wine/sparkling wine (0.1 l) or liquor (0.02 l)≈10 g alcohol*).

#### Drug consumption

Not only alcohol consumption has psychological side effects, but also drug consumption. A survey of over 20,000 people showed that participants with a mental illness have a higher probability of developing substance abuse as well. Conversely, substance addicts have a seven-fold increased likelihood of developing a mental disorder. The highest comorbidity rate was found in drug addiction disorders (excluding alcohol addiction), of which more than half (53%) also had a mental disorder [35]. The following substances were queried to record drug consumption: Opiates (e.g., heroin, methadone), THC (cannabis), smoke mixtures (e.g., Spice), strong pain relievers (e.g. Tramal, Valoron), sedatives, sleeping pills (e.g. Benzos), cocaine, amphetamines (e.g. Crystal Speed), ecstasy, hallucinogens (e.g. LSD, mushrooms), "brain enhancers" (e.g. Ritalin, Modafinil, and methylphenidate), legal highs (e.g. bath salts) and other substances. The following possible answers for drug consumption were given: *no consumption; consumption not last year, consumption not in the last 30 days; consumption last 30 days 1 x; use more often in the last 30 days; daily use for the last 30 days*. A drug index was created for the regression analyses. For this purpose, the values ​​of the individual variables were added up for the twelve different drugs. Correspondingly, values ​​between 12 (no consumption at all) and 72 (daily consumption of all substances) can be assumed.

#### Body-Mass-index (BMI)

The BMI was calculated using the person´s height and weight. For further calculations, the following categories were created based on the recommendations of the World Health Organization on the BMI: underweight: < 18.50; normal weight: 18.50 to 25.00; overweight: 25.01-29.99; obesity: > 30.00 [36]. This categorical variable was also dummy coded with normal weight as the reference category.

#### Smoking

In order to measure the smoking behavior, the number of cigarettes smoked per day was asked. Since the non-smokers were not included in this question, this group was added in SPSS by defining the value 0 (zero cigarettes per day) for the non-smokers. 

## Results

### Sample characteristics

A sample of 1,842 students took part in the online survey, which corresponds to a response rate of 11.4%. The average age of the participants was just under 25 years (*SD* = 4.3), 52.1% of the students were male and 47.3% female (11 students did not state their gender). The following Table 1 shows statistical parameters for the variables that were used for the regression analyses.

**Table 1:** Characteristics of the sample

Variables	M	Me	SD	Mn	Mx	Ms
**Age in years**	25.3	24.9 (IQR = 4.0)	4.53	18.0	54.0	20
**Body Mass Index**	22.95	22.58 (IQR = 3.39)	3.25	12.35	51.9	18
**Smoking** (number of cigarettes per day)	1.77	0.0 (IQR = 0)	4.83	0.00	60.0	64
**Physical activity** (hours of sport per week)	3.91	3.00 (IQR = 5.0)	3.80	0.00	40.0	177
**Nutritional behavior** (0 = ”unhealthy”; 4 = ”healthy”)	2.56	3.00 (IQR = 0.75)	0.74	1.00	4.00	13
**Alcohol consumption** (in gram)	24.64	25.00 (IQR = 20.0)	18.55	0.00	180.0	52
**Drug consumption** (12 = no consumption; 72 = daily consumption of all listed drugs)	13.63	12.00 (IQR = 2.0)	3.77	12.0	72.0	66
**Mental HRQOL** (-1.266 = lowest mental HRQOL; 80.739 = highest mental HRQOL)	46.67	49.90 (IQR 12.25)	10.43	6.00	67.51	28
**Depression** (0 = not depressed; 37 = very depressed)	12.56	11.0 (IQR = 8)	6.20	0.00	37.00	41
**Anxiety** (4 = strong, 3 = moderate, 2 = hardly, 1 = not at all)	1.39	1.00 (IQR = 1)	0.72	1.00	4.00	11

*M* Arithmetic mean, *Me* Median, *SD* Standard deviation, *Mn* Minimum, *Mx* Maximum, *Ms* Missing values

### Gender differences

Table 2 shows the mean comparison test with standard deviation and significant differences between gender. Every variable, except for age, showed significant differences between female and male students.

**Table 2:** Variables of analyses stratified by gender (T-Test)

		*n*	M	SD	p
**Age**	male	*947*	**25.16**	4.04	.059
	female	*868*	**24.76**	4.98	
**Faculty: Economy**	male	*857*	**.73**	.44	.000
	female	*776*	**.28**	.45	
**Faculty: Technical**	male	*857*	**.22**	.41	.000
	female	*776*	**.41**	.49	
**Underweight**	male	*943*	**.01**	.11	.000
	female	*860*	**.08**	.28	
**Overweight**	male	*943*	**.71**	.45	.005
	female	*860*	**.77**	.42	
**Adipositas**	male	*943*	**.24**	.43	.000
	female	*860*	**.12**	.33	
**BMI**	male	*943*	**23.88**	3.12	.000
	female	*861*	**21.98**	3.27	
**Nutrition Behaviour**	male	*948*	**1.93**	.63	.000
	female	*868*	**2.48**	.67	
**Physical Activity**	male	*892*	**4.52**	4.02	.000
	female	*759*	**3.18**	3.39	
**Smoking**	male	*916*	**2.01**	5.31	.025
	female	*849*	**1.50**	4.20	
**Alcohol consumption**	male	*939*	**28.10**	20.53	.000
	female	*837*	**21.02**	16.07	
**Drug consumption**	male	*928*	**14.05**	4.59	.000
	female	*837*	**13.25**	3.22	

There were also differences between women and men concerning the nutritional behaviour, with more female students eating healthily or even very healthily (60%) while it is only 34.6% for men. A total of 65.4% of men ate “unhealthy” food, whereas only 33.1% of female students were in this category.

21.8% of the students stated that they do not participate in any sports, in average as many men as women. In contrast, women more often than men stated that they do one to three hours of sports per week, whereas male students were more likely to be represented in the category of four to six hours of sports per week. In fact, there were more than twice as many men than women who did seven to ten hours or more than ten hours of exercise a week. To test whether the differences are statistically significant, an chi-squared test test was carried out. The relation between these variables was significant, χ^2^ (4, *N* = 1650) = 83.8, *p* < 0.001.

A total of 15.1% of the students stated that they did not drink alcohol; when differentiated according to gender, this was 12.4% for men and 18.2% for women. Very few male students (2.9%) drank up to 10 g of alcohol per day, while almost six times as many female students, namely 17.2%, fall into this category. 68.6% of men and 43% of women drank more than 20 g of alcohol per day. Considering the recommended amount ​​for alcohol consumption per day, only 31.5% of men were within the range of the recommendation of a maximum of 20 g per day. Among women, 35.4% were in the range of the maximum recommended amount of no more than 10 g of alcohol per day.

With the 30-day prevalence, THC was most frequently represented with 16.8% in male and 7.1% in female students. This was followed by cocaine for men with 2.2% and pain relievers, sedatives and sleeping pills for women with 1.5% each. Regarding to smoking status, 18.5% of the students reported that they do smoke.

### Mental Health-Related Quality of Life (HRQOL)

**Fig. 1:**
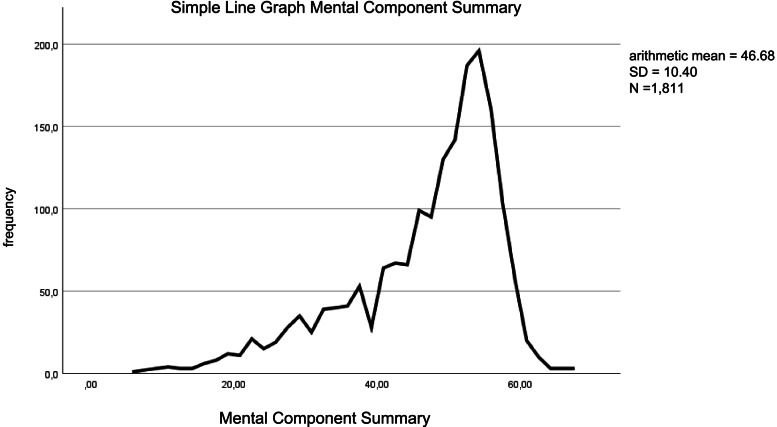
Simple Line Graph of the Mental HRQOL (MCS)

The means ​​of mental HRQOL were below the values for a German or American norm sample. Overall, in our sample, the mean was 46.68 (SD = 10.40). The following Fig. 1 shows a simple line diagram of the mental HRQOL.

**Fig. 2:**
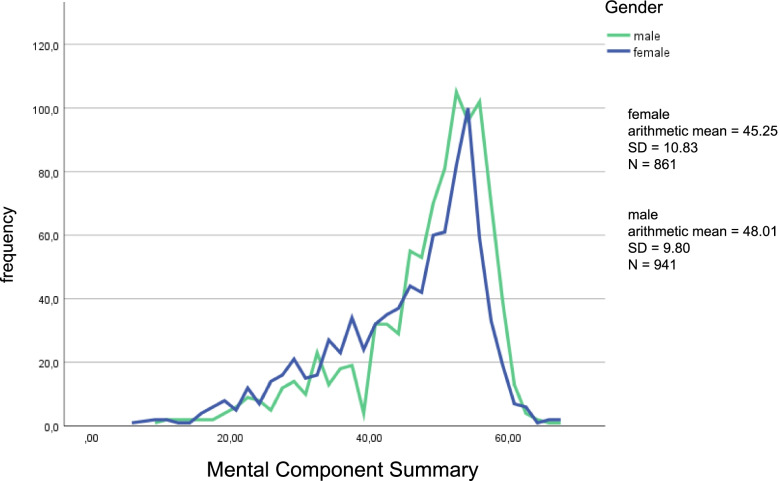
Line Graphs of the mental HRQOL (MCS) differentiated by gender

Differentiated by gender, the mental HRQOL for women reached an arithmetic mean of 45.25 (SD = 10.83) and for men 48.01 (SD = 9.80). Thus the values ​​are again below those of the norm sample [37]. The following Fig. 2 shows a line diagram of the mental HRQOL, whereby a distinction was made between male and female students.

When it comes to anxiety, there were again clear gender differences. More than twice as many women stated that they suffer from severe or moderate anxiety, i.e., a total of 13.6% of female students and 6.4% of male students. 79.4% of men and 64.5% of women had no restrictions due to fears.

### Multiple linear regression analyses

In order to test the multicollinearity in advance, a correlation matrix was created with all independent variables in order to be able to check the correlation coefficients. No multicollinearity could be determined because there were no correlations above *r* = 0.80. The highest significant correlation coefficient was *r *= 0.400** (*p* < 0.01) smoking and drug use). Table 3 shows the correlation coefficients of the independent variables and their significances.

**Table 3:** Test on Multicollinearity

	**Gender**	**Age**	**Faculty**	**BMI**	**Nutrition**	**Physical activity**	**Smoking**	**Alcohol**	**Drugs**
**Gender**	1	-.044	.461	-.190**	.388**	-.176**	-.053*	-.186**	-.098**
**Age**	-.044	1	.145**	.165**	.082**	-.065**	.079**	-.019	.023
**Faculty**	.461**	.145**	1	-.063*	.258**	-.141**	.027	-.059*	.001
**BMI**	-.190**	.165**	-.063*	1	-.153**	-.030	.062**	.059*	-.024
**Nutrition**	.388**	.082**	.258**	-.153**	1	.053*	-.111**	-.218**	-.050*
**PA**	-.176**	-.065**	-.141	-.030	.053*	1	-.099**	.021	-.006
**Smoking**	-.053*	.079**	.027	.062*	-.111**	-.099**	1	.289**	.400**
**Alcohol**	-.186**	-.019	-.059*	.059*	-.218**	.021	.289**	1	.373**
**Drugs**	-.098**	.023	.001	-.024	-.050*	-.006	.400**	.373**	1

Significance levels: * *p* < 0.05, ** *p* < 0.01, ** *p* < 0.001

*BMI* Body Mass Index, *PA* Physical Activity

### Multiple regression analyses of mental health-related quality of life, depression and anxiety

As expected, gender was a significantly associated with mental health indicators. Physical activity and alcohol (only for female students) were respectively positively associated with mental HRQOL, depression, and anxiety. Smoking, drug use and being underweight (only for male students) were negatively associated with mental HRQOL. Drug consumption had the greatest effect, followed by gender, smoking, physical activities and finally alcohol consumption. Table 4 below includes the standardized beta values ​of the variables with significances from all nine regression models.

**Table 4:** Multiple regression analyses of mental HRQOL, depression, and anxiety

	**Dependent Variables**								
**Independent variables**	**Mental HRQOL (total)**	**Mental HRQOL (male)**	**Mental HRQOL (female)**	**Depression (total)**	**Depression (male)**	**Depression (female)**	**Anxiety (total)**	**Anxiety (male)**	**Anxiety (female)**
	β	β	β	β	β	β	β	β	β
Gender	-.094**	**-**	**-**	.137***	**-**	**-**	.169***	**-**	**-**
Age	-.051 n.s	-1.05**	.000 n.s	.064*	.105**	.023 n.s	.014 n.s	.102**	-.059 n.s
Faculty^a^									
Economy	.044 n.s	.089	.023 n.s	-.044 n.s	-.086 n.s	-.026 n.s	-.017 n.s	-.070 n.s	.016 n.s
Technology	.053 n.s	.099	.039 n.s	-.053 n.s	-.096 n.s	-.004 n.s	-.039 n.s	-.068 n.s	-.037 n.s
BMI^b^									
Underweight	-.027 n.s	-.087*	.008 n.s	.033 n.s	.079 n.s	-.004 n.s	.013 n.s	.055 n.s	-.077 n.s
Overweight	.014 n.s	.004 n.s	-.003 n.s	-.054 n.s	-.046 n.s	-.046 n.s	.017 n.s	.106 n.s	-.083 n.s
Adipositas	.025 n.s	.016 n.s	.001 n.s	-.065 n.s	-.053 n.s	-.054 n.s	-.052 n.s	.038 n.s	-.150 n.s
Nutrition	.015 n.s	.008 n.s	.007 n.s	-.034 n.s	-.029 n.s	-.025 n.s	-.005 n.s	-.026 n.s	.038 n.s
Physical activity	.085**	.093*	.080 n.s	-.101***	-.127**	-.067 n.s	-.027 n.s	-.012 n.s	-.047 n.s
Smoking	-.084**	-.102*	-.049 n.s	.101**	.122**	.061 n.s	.108***	.137**	.092 n.s
Alcohol	.066*	.036 n.s	.121**	-.055 n.s	-.001 n.s	-.149**	-.036 n.s	-.023 n.s	-.065 n.s
Drugs	-.154***	-.103*	-.246***	.166***	.111**	.275***	.109***	.059 n.s	.171**
R^2^	.065	.061	.071	.094	.085	.093	.066	.045	.058
Adjusted R^2^	.056	.046	.053	.086	.070	.075	.057	.030	.040

β = adjusted Beta values; *p < 0.05, **p < 0.01, ***p < 0.001, n.s. = non significant; ^a^ reference: Social Studies, ^b^ reference: normal weight; R^2^ = coefficient of determination

## Discussion

### Principal findings

**Fig. 3:**
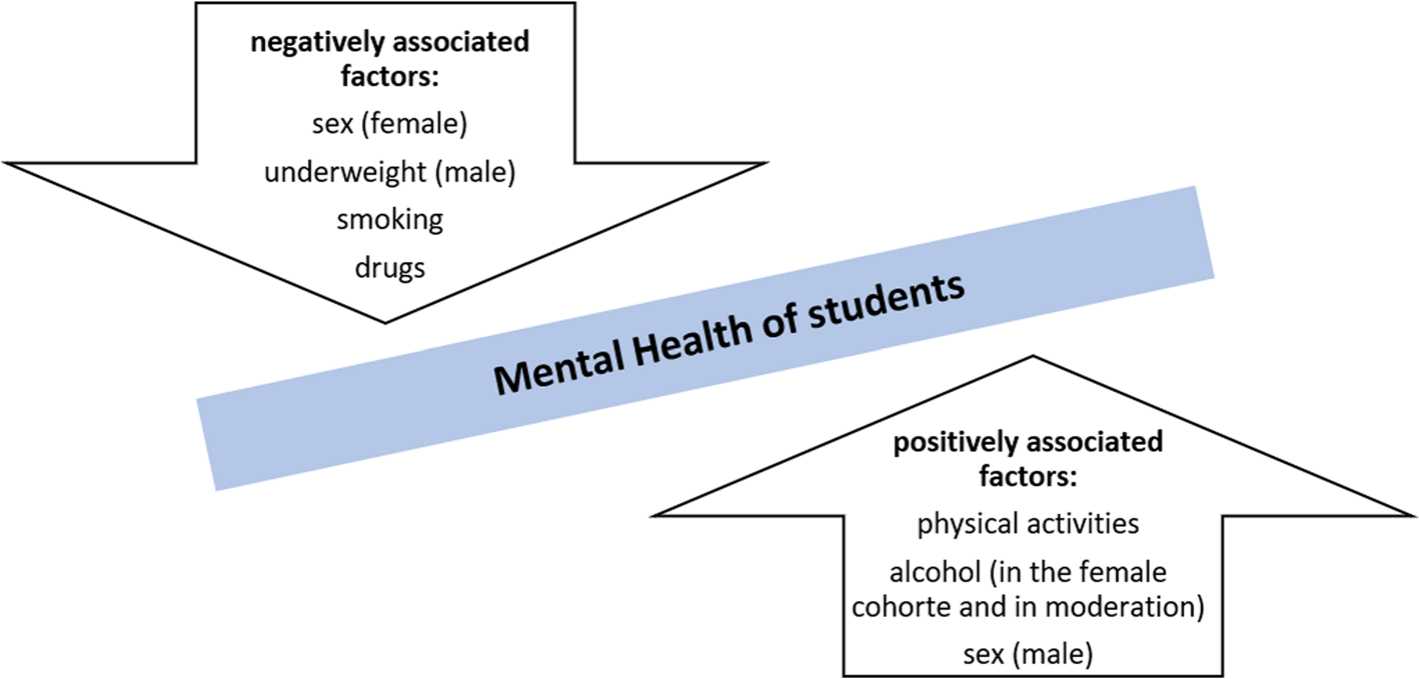
Overview of positively and negatively associated factors for mental health

The aim of the present work was to evaluate the mental health situation in a sample of German university students, as well as to identify associations between lifestyle and sociodemographic factors and the mental health indicators of students. Overall, several lifestyle and sociodemographic factors had significant effects on mental health indicators: gender, age, underweight, drug consumption were positively associated with mental health indicators, while and alcohol (in moderation) and physical activity were negatively associated. In our models, associations between lifestyle and sociodemographic factors and mental health outcomes varied according to gender. Only drug consumption was a significantly associated in both genders. For women, alcohol and drug consumption were significantly associated, while for men, underweight, physical activity, smoking, age and drug consumption were significantly associated. The following Fig. 3 gives an overview of positively and negatively associated factors for mental health for students.

### Comparison with other studies

As expected, gender significantly affected mental health indicators. The regression models confirm the gender difference that has already been identified through the literature research. Women report a lower state of mental health in comparison to men. However, the question arises again as to whether women are actually in a worse state of health than men and are exposed to more risk factors (such as violence or hormonal influences from the menstrual cycle) [19] or whether women are simply more sensitive to their health perception [38]. The causes of the difference in mental health cannot be clarified with the help of this work, but there is no doubt that it makes sense to differentiate the mental health status by gender.

In our sample, smoking was negatively associated with the mental health of male students. Many smokers reported that cigarette consumption reduces stress. Smokers had a fundamentally higher stress level than non-smokers. People who developed a regular smoking behavior reported increased stress levels. In addition, it was shown that smokers reported a normal mood while consuming cigarettes, while the mood deteriorates between cigarettes. Therefore, addicted smokers need nicotine to feel normal. As a result, the relaxing effect of a cigarette merely represents the reverse of the tension and irritation of nicotine withdrawal [39]. It is reasonable that the increased stress level caused by cigarette consumption has a negative impact on mental health. However, the effect was not significant in the female students.

Surprisingly, the consumption of alcohol was positively associated with the mental HRQOL of women and depression. Studies showed that alcohol use can also have positive effects. For example, a small dose of alcohol can have a positive effect on sleep behaviour [40]. In a previous study, the effects of alcohol on the psyche and cognitive abilities were examined. For this purpose, the participants were divided into three groups: 0 g, 8 g (small amount) and 24 g (high amount) alcohol. They found that a small amount of alcohol, unlike no alcohol, improved the results of the cognitive test and had positive effects on mood (reduced tension, insecurity and restlessness). In addition, alcohol lifts the mood and has a rewarding effect on people [41]. Researchers investigated the nature of the effects of alcohol on the brain and found that the endogenous opioids produced by the body were increased by the consumption of ethanol [42]. Therefore, it is possible that at least small amounts of alcohol do not negatively influence mental health, but on the contrary have a positive effect through the relaxing, mood-lifting and loosening effect. However, this result should be interpreted very cautiously. The long-term effects of alcohol and the harmful consequences of excessive consumption are not to be neglected. The question remains open whether higher alcohol consumption leads to better mental health or whether, conversely, students with better mental health drink more alcohol. It would be very interesting to conduct a longitudinal analysis to obtain answers to this question.

The consumption of drugs, on the other hand, was negatively associated with mental health, in both men and women. When it comes to illegal drug consumption, it is also noticeable that women consume all substances less than men with the exception of painkillers and sedatives. Thus, are women more likely to experience pain or are they more sensitive to it? Young women were asked about their menstrual symptoms from which 84.1% stated that they had pain during their menstruation and 43.1% even reported menstrual pain with each cycle. As a result, 31.9% of women state that they are dependent on pain medication. Menstrual pain also affects the quality of life and worsens school performance. In addition, 44.6% of women with menstrual pain had impaired social skills [43]. This could explain why women take more pain medication and why their mental health was reported worse.

### General state of health-related factors

In our study, 81.5% of the students stated that they do not smoke, which is higher than the average of the total German population (77,6%) [44], and also higher compared to the total of young German adults aged 18–25 (75,2%) [45]. This study also found a correlation between education and smoking: the higher the level of education, the fewer the smokers. This could be an explanation for the high proportion of non-smokers in the present survey of students. 68.6% of male students and 43% of female students reported alcohol consumption that is classified as unhealthy according to the recommendations of the DGE [46]. In comparison, the values for risky alcohol consumption are much lower in a study about the alcohol consumption of young adults in Germany (18–25 years). According to this study, 20.9% of the men and 15.4% of the women had risky alcohol consumption [47]. However, it must be considered that the study was based on higher threshold values; risky alcohol consumption was recorded at 24 g of pure alcohol per day for men and 12 g of pure alcohol per day for women.

Students are generally seen by society as a healthy cohort who do not need any special preventive support, although they could certainly be classified as a disadvantaged group of people due to various factors, such as low income or poor housing and working conditions [48]. A comparison of the mental HRQOL with the German and American norm samples shows that students are slightly below the average values of the norm samples and not—as society assumes—at least equal, if not better.

### Limitations

There are limitations concerning the nutrition index used in the analyses. The question that arises is: What can be defined as healthy eating? Can this be defined as the consumption of a lot of vegetables or fruit and little meat or sausage or is healthy eating describing the mediterranean diet? It is probably difficult to define a single healthy diet. In the previous literature research, healthy eating was interpreted differently [28–30]. It is therefore questionable whether consuming little meat, due to certain nutrients in meat, is healthier than consuming no meat at all. Significantly more women fell into the healthy eating category than men. However, it is possible that precisely these women are pathologically trying to eat “healthily”, and thus an eating disorder can stand behind it. In recent years, in addition to eating disorders such as bulimia, anorexia or binge eating, orthorexia nervosa has also become more prevalent. Affected people are obsessed with eating “healthily” and therefore avoid favorite foods. It is no longer about the joy of eating, but about the health value of the food consumed, which is why the food is steadily reduced and in the end, mainly fruit and vegetables are consumed [49]. With the calculated nutrition index, the students affected by orthorexia nervosa could have been identified. A systematic review with data from 67 studies showed that significantly more women were affected by orthorexia nervosa than men [50]. In addition, female students' obsession with consuming healthy food correlated with the worry of being overweight, the importance of external appearance, fitness orientation, health orientation and satisfaction with appearance [51]. It could be that the nutritional index declares pathological eating behavior to be positive and thus influences the result. Limitations can also be found in other indices. For depression and anxiety, for example, no standardized, validated instruments were used, but own indices were formed, based on standardized instruments.

### Generalization of the findings

The sample used was a full survey at the Munich University of Applied Sciences. A generalization of the results for students in general seems rather difficult, because only students from Munich took part in the survey. Some subgroups were probably underrepresented in our data set, such as those with lower socioeconomic status or mental health issues. In addition, we presume that we primarily studied German-citizen students. Students from different countries were assumedly hard to reach. Presumably, the students in our sample were more privileged and healthier, which may understate the associations between lifestyle factors and mental health indicators in deprived subgroups. Furthermore, social desirability bias may have affected the statistics. Students with lower socioeconomic status or mental health issues may be more likely to be impacted. An overarching survey at several universities would be more suitable for this. We expect that our findings to be transferable to universities of applied sciences in high-income countries in Europe. Our findings are a valuable contribution to university students´ mental health. However, to increase or maintain students mental health status associated factors need to be further investigated.

## Conclusion

In conclusion, it can be seen that students score worse than the German norm sample in terms of mental HRQOL. It is advisable to detect mental health problems as early as possible and to counteract them in order to avoid secondary diseases. Even before the COVID-19 pandemic, mental health problems were already more prevalent among students. Due to the additional mental stress caused by the pandemic, it can be assumed that mental health problems have even increased. Thus, higher education institutions should pay more attention to the mental health of their students.

With regard to mental health, it is advantageous to look at female and male students differently. This could be an important finding for psychological counselling centers at universities, which should adapt their counselling strategies and methods accordingly. With regard to this, universities must be made more aware of the need to take the first signs seriously. It is important that the counselling centers are aware of the risk and protective factors of mental illnesses in order to be able to make appropriate recommendations, such as regular exercise. This implies a close cooperation between research and counselling centers. Counselling centers must always be up-to-date with the latest research so they can adapt and align their methods and offers accordingly. It is therefore advisable to accelerate further research of the risk and protective factors for students’ mental health. In particular, protective factors should be researched more extensively. It would be interesting to analyse the mental health of students after the COVID-19 pandemic and compare it with the state before the pandemic.

## Supplementary Information

**Additional file 1.**

## Data Availability

All data generated or analysed during this study are included in this published article and its supplementary information files.

## References

[1] Statista. Anzahl der Studierenden an Hochschulen in Deutschland in den Wintersemestern von 2002/2003 bis 2020/2021. 2021. https://de.statista.com/statistik/daten/studie/221/umfrage/anzahl-der-studenten-an-deutschen-hochschulen/#professional. Accessed 13.05.21.

[2] Fegert JM, Hauth I, Banaschewski T, Freyberger HJ (2017). Übergang zwischen Jugend- und Erwachsenenalter: Herausforderungen für die Transitionspsychiatrie. Z Kinder Jugendpsychiatr Psychother.

[3] Bundesministerium für Bildung und Forschung. Die wirtschaftliche und soziale Lage der Studierenden in Deutschland 2016: 21. Sozialerhebung des Deutschen Studentenwerks durchgeführt vom Deutschen Zentrum für Hochschul- und Wissenschaftsforschung. 2017. http://www.sozialerhebung.de/download/21/Soz21_hauptbericht.pdf. Accessed 26 Jun 2018.

[4] Son C, Hegde S, Smith A, Wang X, Sasangohar F (2020). Effects of COVID-19 on College Students' Mental Health in the United States: Interview Survey Study. J Med Internet Res.

[5] Herrman H, Saxena S, Moodie R (2005). Promoting Mental Health: Concepts Emerging Evidence Practice.

[6] Rief W, First MB (2017). Handbuch der Differenzialdiagnosen - DSM-5®.

[7] Assari S. Multiplicative Effects of Social and Psychological Risk Factors on College Students' Suicidal Behaviors. Brain Sci 2018. 10.3390/brainsci8050091. 10.3390/brainsci8050091 PMC5977082 29772772

[8] El Ansari W, Stock C, Snelgrove S, Hu X, Parke S, Davies S (2011). Feeling healthy? A survey of physical and psychological wellbeing of students from seven universities in the UK. Int J Environ Res Public Health.

[9] Barthel Y, Ernst J, Rawohl S, Körner A, Lehmann A, Brähle E (2011). Psychosoziale Situation von Studierenden – Beratungs und Behandlungsbedarf und Interesse an Psychotherapie. ZPPM Zeitschrift für Psychotraumatologie, Psychotherapiewissenschaft, Psychologische Medizin.

[10] Holm-Hadulla RM, Hofmann F-H, Sperth M, Funke J (2009). Psychische Beschwerden und Störungen von Studierenden. Psychotherapeut.

[11] Office of Research on Women´s Health. Sex & Gender. 2021. https://orwh.od.nih.gov/sex-gender. Accessed 5 Sep 2021.

[12] Leombruni P, Corradi A, Lo Moro G, Acampora A, Agodi A, Celotto D (2022). Stress in Medical Students: PRIMES, an Italian, Multicenter Cross-Sectional Study. Int J Environ Res Public Health.

[13] Bailer J, Schwarz D, Witthöft M, Stübinger C, Rist F (2008). Prävalenz psychischer Syndrome bei Studierenden einer deutschen Universität. [Prevalence of mental disorders among college students at a German university]. Psychother Psychosom Med Psychol.

[14] Vankim NA, Nelson TF (2013). Vigorous physical activity, mental health, perceived stress, and socializing among college students. Am J Health Promot.

[15] Velten J, Bieda A, Scholten S, Wannemüller A, Margraf J (2018). Lifestyle choices and mental health: a longitudinal survey with German and Chinese students. BMC Public Health.

[16] Wörfel F, Gusy B, Lohmann K, Töpritz K, Kleiber D (2016). Mental health problems among university students and the impact of structural conditions. J Public Health.

[17] Jacobi F, Höfler M, Strehle J, Mack S, Gerschler A, Scholl L (2014). Psychische Störungen in der Allgemeinbevölkerung: Studie zur Gesundheit Erwachsener in Deutschland und ihr Zusatzmodul Psychische Gesundheit (DEGS1-MH). [Mental disorders in the general population : Study on the health of adults in Germany and the additional module mental health (DEGS1-MH)]. Nervenarzt.

[18] Bert F, Ferrara M, Boietti E, Langiano E, Savatteri A, Scattaglia M (2022). Depression, Suicidal Ideation and Perceived Stress in Italian Humanities Students: A Cross-Sectional Study. Psychol Rep.

[19] World Health Organization. Mental health: new understanding, new hope. 2001. https://apps.who.int/iris/handle/10665/42390. Accessed 24 Jun 2022.

[20] Greim L (2014). Gesundheitsbezogene Lebensqualität der Studierenden an der Hochschule München: Ergebnisse einer quantitativen Fragebogenerhebung als Chance für eine Gesundheitsfördernde Hochschule [unpublished Master Thesis].

[21] Field A (2013). Discovering statistics using IBM SPSS statistics: And sex and drugs and rock 'n' roll.

[22] Wood AM, Kaptoge S, Butterworth AS, Willeit P, Warnakula S, Bolton T (2018). Risk thresholds for alcohol consumption: Combined analysis of individual-participant data for 599 912 current drinkers in 83 prospective studies. Lancet.

[23] Morfeld M, Kirchberger I, Bullinger M. SF-36 - Fragebogen zum Gesundheitszustand Deutsche Version des Short Form- 36 Health Survey. 2011. https://www.testzentrale.de/shop/fragebogen-zum-gesundheitszustand.html. Accessed 24 Jun 2022.

[24] Beck AT, Steer RA, Brown G (1996). PsycTESTS Dataset.

[25] von Zerssen D, Petermann F (2011). B-LR Beschwerden-Liste: Revidierte Fassung.

[26] Aceijas C, Waldhäusl S, Lambert N, Cassar S, Bello-Corassa R (2017). Determinants of health-related lifestyles among university students. Perspect Public Health.

[27] Antonopoulou M, Mantzorou M, Serdari A, Bonotis K, Vasios G, Pavlidou E (2020). Evaluating Mediterranean diet adherence in university student populations: Does this dietary pattern affect students' academic performance and mental health?. Int J Health Plann Manage.

[28] Lo Moro G, Corezzi M, Bert F, Buda A, Gualano MR, Siliquini R. Mental health and adherence to Mediterranean diet among university students: an Italian cross-sectional study. J Am Coll Health. 2021:1–11. 10.1080/07448481.2021.1970567. 10.1080/07448481.2021.1970567 34519625

[29] Mikolajczyk RT, El Ansari W, Maxwell AE (2009). Food consumption frequency and perceived stress and depressive symptoms among students in three European countries. Nutr J.

[30] Saghafian F, Malmir H, Saneei P, Keshteli AH, Hosseinzadeh-Attar MJ, Afshar H (2018). Consumption of fruit and vegetables in relation with psychological disorders in Iranian adults. Eur J Nutr.

[31] Blumenthal JA, Babyak MA, Doraiswamy PM, Watkins L, Hoffman BM, Barbour KA (2007). Exercise and pharmacotherapy in the treatment of major depressive disorder. Psychosom Med.

[32] Klosterkötter J, Maier W (2003). Früherkennung und Frühintervention bei psychischen Störungen: Ansätze zur Prävention und zur Vermeidung von Chronifizierungen. Deutsches Ärzteblatt.

[33] Kushner MG, Sher KJ, Erickson DJ (1999). Prospective analysis of the relation between DSM-III anxiety disorders and alcohol use disorders. Am J Psychiatry.

[34] Deutsche Gesellschaft für Ernährung e.V. Alkoholkonsum – welche Mengen sind gesundheitlich verträglich? 2018. https://www.dge.de/presse/pm/alkoholkonsum-welche-mengen-sind-gesundheitlich-vertraeglich/. Accessed 13.05.21.

[35] Regier DA (1990). Comorbidity of mental disorders with alcohol and other drug abuse. Results from the Epidemiologic Catchment Area (ECA) Study. JAMA.

[36] WHO. Health Topics: Body mass index - BMI. 2019. http://www.euro.who.int/en/health-topics/disease-prevention/nutrition/a-healthy-lifestyle/body-mass-index-bmi. Accessed 29.0.2019.

[37] Ellert U, Kurth B-M (2004). Methodische Betrachtungen zu den Summenscores des SF-36 anhand der erwachsenen bundesdeutschen Bevölkerung. Bundesgesundheitsblatt Gesundheitsforschung Gesundheitsschutz.

[38] Ek S (2015). Gender differences in health information behaviour: a Finnish population-based survey. Health Promot Int.

[39] Parrott AC (1999). Does cigarette smoking cause stress?. Am Psychol.

[40] Stone BM (1980). Sleep and low doses of alcohol. Electroencephalogr Clin Neurophysiol.

[41] Lloyd HM, Rogers PJ (1997). Mood and cognitive performance improved by a small amount of alcohol given with a lunchtime meal. Behav Pharmacol.

[42] Mitchell JM, O’Neil JP, Janabi M, Marks SM, Jagust WJ, Fields HL (2012). Alcohol consumption induces endogenous opioid release in the human orbitofrontal cortex and nucleus accumbens. Sci Transl Med.

[43] Grandi G, Ferrari S, Xholli A, Cannoletta M, Palma F, Romani C (2012). Prevalence of menstrual pain in young women: what is dysmenorrhea?. J Pain Res.

[44] Statistisches Bundesamt. Gesundheitszustand in Deutschland und -relevantes Verhalten. 2019. https://www.destatis.de/DE/Themen/Gesellschaft-Umwelt/Gesundheit/Gesundheitszustand-Relevantes-Verhalten/_inhalt.html. Accessed 24 Jun 2022.

[45] Orth B, Merkel C (2019). Rauchen bei Jugendlichen und jungen Erwachsenen in Deutschland. Ergebnisse des Alkoholsurveys 2018 und Trends.

[46] Deutsche Gesellschaft für Ernährung e.V. Alkoholkonsum ‐ welche Mengen sind gesundheitlich verträglich? 2018.

[47] Orth B, Merkel C (2019). Der Alkoholkonsum Jugendlicher und junger Erwachsener in Deutschland. Ergebnisse des Alkoholsurveys 2018 und Trends: BZGA - Federal Centre for Health Education.

[48] Stock C, Krämer A. „Wie gesund leben Studierende? - Schlussfolgerungen für eine gesundheitsfördernde Hochschule“. In: Badura B, Litsch M, Vetter C, editors. Gesundheitsmanagement im öffentlichen Sektor. Berlin, Heidelberg, New York, Barcelona, Hongkong, London, Mailand, Paris, Tokio: Springer; 2002. p. 180–194. 10.1007/978-3-642-56022-4_13.

[49] Kinzl JF, Hauer K, Traweger C, Kiefer I (2005). Orthorexia nervosa: Eine häufige Essstörungbei Diätassistentinnen?. Ernährungs-Umschau.

[50] Strahler J (2019). Sex differences in orthorexic eating behaviors: a systematic review and meta-analytical integration. Nutrition.

[51] Brytek-Matera A, Donini LM, Krupa M, Poggiogalle E, Hay P (2015). Orthorexia nervosa and self-attitudinal aspects of body image in female and male university students. J Eat Disord.

